# lme4GS: An R-Package for Genomic Selection

**DOI:** 10.3389/fgene.2021.680569

**Published:** 2021-06-18

**Authors:** Diana Caamal-Pat, Paulino Pérez-Rodríguez, José Crossa, Ciro Velasco-Cruz, Sergio Pérez-Elizalde, Mario Vázquez-Peña

**Affiliations:** ^1^Department of Socioeconomics, Statistics, and Informatics, Colegio de Postgraduados, Texcoco, Mexico; ^2^Biometrics and Statistics Unit, International Maize and Wheat Improvement Center (CIMMYT), Texcoco, Mexico; ^3^Department of Irrigation, Universidad Autónoma Chapingo, Texcoco, Mexico

**Keywords:** genomic selection, genomic prediction, linear mixed model, lme4, kernel

## Abstract

Genomic selection (GS) is a technology used for genetic improvement, and it has many advantages over phenotype-based selection. There are several statistical models that adequately approach the statistical challenges in GS, such as in linear mixed models (LMMs). An active area of research is the development of software for fitting LMMs mainly used to make genome-based predictions. The lme4 is the standard package for fitting linear and generalized LMMs in the R-package, but its use for genetic analysis is limited because it does not allow the correlation between individuals or groups of individuals to be defined. This article describes the new lme4GS package for R, which is focused on fitting LMMs with covariance structures defined by the user, bandwidth selection, and genomic prediction. The new package is focused on genomic prediction of the models used in GS and can fit LMMs using different variance–covariance matrices. Several examples of GS models are presented using this package as well as the analysis using real data.

## Introduction

With the new, low-cost, high-throughput genotyping technologies of the last decade, a breeding selection paradigm called genomic selection (GS) has emerged (Meuwissen et al., 2001). GS combines molecular and phenotypic data to obtain the genomic estimated breeding values (GEBVs) of individuals that have been genotyped but not phenotyped (Bernardo and Yu, 2007; de los Campos et al., 2009; Hayes et al., 2009; VanRaden et al., 2009; Crossa et al., 2010). The main advantages of GS over family-based selection in breeding are that it reduces the cost per cycle and the time required for variety development. However, several factors could impact the accuracy of prediction; they occur at different levels and are influenced by several genetic, environmental, and statistical factors.

Complications arise in GS when determining (i) the size and diversity of the training population, (ii) the relationship between the training and testing sets, (iii) genetic complexity, and (iv) the heritability of the traits to be predicted. Challenges in GS are related to the high dimensionality of marker data, where, the number of markers is much larger than the number of observations, the multi-collinearity among markers, the cryptic interaction between markers, the complexity of the trait, sample size, correlation among markers, and the ever-present genotype × environment interaction. These complexities require parametric and semi-parametric statistical models, especially mixed models, Bayesian estimations, and, recently, deep machine learning methods that can deal appropriately with the usually large datasets (Crossa et al., 2017). This has led to computational challenges due to the data size and statistical challenges that include model fitting and parameter optimization. Therefore, the development of complete and simple computer packages to estimate the GEBV of the individuals to be selected under these complex scenarios is crucial for an efficient application of GS.

The first R software (R Core Team, 2021) developed for genome-based prediction was presented by de los Campos et al. (2009). Shortly afterward, Pérez et al. (2010) formally described the Bayesian linear regression (BLR) that allows fitting high-dimensional linear regression models including dense molecular markers, pedigree information, and several other covariates other than markers. The BLR R-package described by Pérez et al. (2010) allows including not only markers but also pedigree data jointly. Furthermore, Pérez et al. (2010) explained the challenges that arise when evaluating genomic-enabled prediction accuracy through random cross-validation (CV), as well as how to select the best choice of hyperparameters for the Bayesian models.

Linear mixed models play a fundamental role in GS and genomic-enabled predictions. This kind of models is widely used for predictions, although other models, such as nonlinear models, neural networks, and other machine learning models, could be used for this purpose. The standard linear mixed model of the form **y** = **X**β + **Zu** + **e**, where, **y** is a response vector of dimension *n* × 1; **X** and **Z** are the design matrices for the fixed (β) and genotypic random (**u**) effects, respectively; and two variance components are estimated $\text{u}∼MN\left(0,\sigma_{u}^{2}\text{K}\right)$, with **K** being a known semidefinite variance–covariance matrix and $\text{e}∼MN\left(0,\sigma_{e}^{2}\text{I}\right).$ In the context of GS, **K** could be the additive relationship matrix derived from the coefficient of co-ancestry (numerator relationship matrix **A**), or it could be the genomic relationship matrix obtained from markers (**G**). As shown below, there are several alternative ways of expressing the incidence matrix **Z** and the vector of random effects **u** when using the numerical relationship matrix (**A**). Bayesian versions of linear regression models have been extensively developed, and their companion software largely distributed and used for research and extended to more complicated cases, for example, the introduction of genotype × environment interaction incorporating pedigree and environmental covariables (Jarquín et al., 2014).

Endelman (2011) developed the rrBLUP R-package, which is able to fit the basic linear mixed model with two variance components ($\sigma_{\text{u}}^{2}$ and $\sigma_{\text{e}}^{2}$) described before with the maximum likelihood or restricted maximum likelihood (REML) methods. As an extra facility, the rrBLUP computes the Gaussian kernel and the exponential kernel that usually account for small cryptic epistatic effects among the markers. The rrBLUP has a CV algorithm to measure the prediction accuracy of the models and shows rapid solutions of the mixed model equations for moderate-to-intermediate data sizes. More specialized computer software, such as the synbreed of Wimmer et al. (2012) and GEMMA of Zhou and Stephens (2012), were later developed.

Although the previously mentioned genomic software programs solve important genomic prediction problems (e.g., prediction in training and testing sets, CV, and estimation of variance parameters), they are separate software pieces without a unified statistical and computing framework. So from the user’s perspective, having a single package implementing all the models to be fitted will save data preparation time and data analysis time. Thus, Pérez and de los Campos (2014) extended the original BLR R-package developed by Pérez et al. (2010) to a more general R-package, the Bayesian generalized linear regression (BGLR) that offers users a great variety of genomic models and methods in a unified computing software for data analysis. The BGLR is available at CRAN. The BGLR package includes several Bayesian regression models, including parametric variable selection and shrinkage methods, and semi-parametric procedures [Bayesian reproducing kernel Hilbert space (RKHS) regressions]. Many non-genomic applications are implemented as well, and response traits can be continuous or categorical (binary or ordinal). The Bayesian algorithm is based on a Gibbs sampler with scalar updates implemented in efficient routines written in C programming language. Furthermore, the BGLR is the main machinery for adapting other more complex genomic models, for example, the complex phenomenon of genotype × environment interaction including pedigree and environmental covariables (Jarquín et al., 2014). The BGLR is also used for assessing the marker effect × environment interaction of Lopez-Cruz et al. (2015) and for fitting Bayesian ridge regression and the Bayes B, as shown by Crossa et al. (2017), or for using the threshold model for ordinal data as did Montesinos-López et al. (2016), and for running all the Bayesian alphabet models.

Although linear mixed models are important tools for fitting GS models, Covarrubias-Pazaran (2016) mentioned like that current GS software includes only one random effect; and therefore, using genomic prediction for more complicated situations hybrid prediction using additive, dominance, and epistatic effects is not possible under the available models. The authors proposed likelihood-based software for fitting mixed models with multiple random effects that allow the user to specify the variance–covariance structure of random effects. Covarrubias-Pazaran (2016) presented an R-package called sommer for genomic prediction with three algorithms for estimating variance components: average information, expectation–maximization, and efficient mixed model association. Results from sommer were comparable with those of other software, and sommer was faster than its Bayesian counterparts.

The development of software for fitting linear mixed models is an active area of research. The use of pedigree and genomic-enabled prediction linear mixed models is crucial for advancing the application of genomic-assisted breeding. The lme4 package (Bates et al., 2015) for R (R Core Team, 2021) has efficient functions for analyzing linear mixed models and generalized linear mixed models (GLMMs). Some of the main features of lme4 are that (i) it is efficient for large dataset problems; (ii) it handles any number of grouping factors, nested or cross-classified; and (iii) it can use a combination of sparse and dense matrix representations to facilitate the processing of large datasets at high computational speed.

However, the use of lme4 for genetic analysis has been limited because it does not allow using the correlation between individuals or groups of individuals. When individual lines or animals are related, the marginal likelihood must allow using this covariance between relatives. Vazquez et al. (2010) developed a package called pedigreemm that uses the lme4 but allows for correlations between levels of random effects, such as those due to genetic relationships between relatives expressed as pedigree relationships. The methodology of Vazquez et al. (2010) uses the numerator relationship matrix **A** (a positive-definite matrix) and subjects it to the Cholesky decomposition, where, the Cholesky factor (**L**) can be obtained from the pedigree information.

Based on the above considerations and some limitations in terms of the computing efficiency of some existing genomic-enabled prediction models, in this research, we describe the new lme4GS R-Package that is based on the lme4 software of Bates et al. (2015) that is available in CRAN. The lme4GS is focused on genomic-based prediction of GS and can fit mixed models with several different variance–covariance matrices. The lme4GS introduces fixed and random effects, and associated variance–covariance matrices, from which matrices for fixed and random effects (**X**, **Z**_1_, …, **Z**_q_, respectively) are obtained. The original variance–covariance matrices are introduced and transformed by using the Cholesky factorization or the eigenvalue decomposition of variance–covariance matrices and later used for defining the objective function (deviance function). Once the objective function has been defined, the optimization module optimizes the objective function and provides REML estimates of the parameters of interest.

## Materials and Methods

Consider the linear mixed model:

(1) y=Xβ+Zu+e,

where, **y** is a response vector of dimensions *n* × 1, **X** is a matrix of fixed effects of dimensions *n* × *p*, β is a vector of fixed effects of dimensions *p* × 1, **Z** is an incidence matrix of dimensions *n* × *r*, and **u** is a vector of random effects. We assume $\text{u}∼MN\left(0,\sigma_{a}^{2}\text{K}\right)$ and $\text{e}∼MN\left(0,\sigma_{e}^{2}\text{I}\right)$, with **K** a known variance–covariance matrix, and $\sigma_{a}^{2}$ and $\sigma_{e}^{2}$ are variance parameters associated with **u** and **e**, respectively; furthermore, we assume that **u** and **e** are independently distributed. In the case of GS, the variance–covariance matrix can be derived from markers or from pedigree.

The linear mixed model (1) can be rewritten as;

(2) $$\text{y}=\text{X}\beta +{\text{Z}}^{*}{\text{u}}^{*}+\text{e},$$

where, Z* = **ZL**, with **L** obtained from the Cholesky factorization of **K**; alternatively, Z* = **ZΓΛ**^1/2^ with **Γ** and **Λ** the matrices of eigenvectors and eigenvalues, respectively, obtained from the eigenvalue decomposition of **K**, and ${\text{u}}^{*}∼MN\left(0,\sigma_{u}^{2}\text{I}\right)$. Note that Z*u* has the same distribution as **Zu**; that is, ${\text{Z}}^{*}{\text{u}}^{*}{=}^{d}\text{Zu}∼\text{MN}\left(0,\sigma_{a}^{2}{\text{ZKZ}}^{{}^{\prime }}\right)$.

### Best Linear Unbiased Predictions

Once mixed model (2) is fitted, the conditional means of the random effects can be obtained, that is, ${\hat{\text{u}}}^{*}$. The best linear unbiased predictions (BLUPs) for u* are obtained as follows: u^*=σ^u2Z*′V^*-1(y-Xβ^) where, V^*=σ^u2Z*Z*′+σ^e2I, with ${\hat{\sigma }}_{e}^{2},{\hat{\sigma }}_{u}^{2}$ and $\hat{\beta }$ REML estimates of variance parameters and vector of fixed effects, respectively. The conditional means of random effects for the model in equation (1) are obtained as follows: $\hat{\text{u}}=\text{L}{\hat{\text{u}}}^{*}$ if the Cholesky factorization is used, or alternatively, $\hat{\text{u}}=\Gamma \Lambda^{1/2}{\hat{\text{u}}}^{*}$ if the eigenvalue is used.

### Prediction of New Observations

The main goal of GS is to predict new observations (phenotypic values) or simply obtain the BLUPs for random effects not present in the observed data but drawn from the same population as **u** and **e** (Gilmour et al., 2004). Assume that the random vector **u** and matrix **K** are partitioned as follows:

$$\text{u}=\left[\begin{array}{c} {\text{u}}_{1}\\ {\text{u}}_{2} \end{array}\right],\text{K}=\left[\begin{array}{cc} {\text{K}}_{11} & {\text{K}}_{12}\\ {\text{K}}_{21} & {\text{K}}_{22} \end{array}\right],$$

the BLUPs for **u**_2_ are obtained as:

(3) $$E\left({\text{u}}_{2}|{\text{y}}_{1}\right)={\text{K}}_{21}{\text{K}}_{11}^{-1}{\text{u}}_{1}.$$

In a more general case, model (1) can be extended to include more random effects, that is:

(4) $$\text{y}=\text{X}\beta +\sum_{j=1}^{q}{\text{Z}}_{j}{\text{u}}_{j}+\text{e},$$

where, **Z**_*j*_ is a design matrix of random effects, and **u**_*j*_ is a vector of random effects, *j* = 1, …, *q*, where, *q* corresponds to the number of random terms included in the model. We assume that ${\text{u}}_{j}∼MN\left(0,\sigma_{j}^{2}{\text{K}}_{j}\right)$ is independently distributed. Note that model (1) is a special case of model (4) obtained by setting $q=1,\text{Z}={\text{Z}}_{1},\text{u}={\text{u}}_{1},\text{K}={\text{K}}_{1},\sigma_{a}^{2}=\sigma_{1}^{2}$. Based on the same computational strategy used to rewrite model (1) as the model in (2), model (4) can be rewritten as:

(5) $$\text{y}=\text{X}\beta +\sum_{j=1}^{q}{\text{Z}}_{j}^{*}u_{j}^{*}+\text{e}.$$

### Implementation

The lme4GS package is an extension of the lme4 R-package (Bates et al., 2015); lme4GS development was inspired by existing R-packages, pedigreemm (Vazquez et al., 2010) and lme4qtl (Ziyatdinov et al., 2018), which are focused on quantitative trait locus (QTL) mapping association and linkage studies, whereas, lme4GS is focused on the problem of prediction in GS (Meuwissen et al., 2001) with GBLUP-type models, although the models can be applied in other research areas. lme4GS uses the computational engine provided by the well-tested and widely used lme4 package to fit mixed models with a variance–covariance matrix provided by the user. lme4GS can be considered a generalization of existing package rrBLUP (Endelman, 2011) because it is able to fit model (4), whereas, rrBLUP is able to fit model (1). The package also implements some of the models in the sommer package of Covarrubias-Pazaran (2016). lme4GS uses the high-level modular structure of lmer (formula module, objective function module, optimization module, and output module) to fit the models with variance–covariance matrices provided by the user. The formula module allows the specification of fixed and random effects and associated variance–covariance matrices, from which matrices for fixed and random effects (**X**, **Z**_1_, …, **Z**_*q*_, respectively) are obtained. After that, the variance–covariance matrices are introduced by computing transformed incidence matrices (${\text{Z}}_{j}^{*},j=1,\ldots ,q$) using the Cholesky or eigenvalue decomposition of variance–covariance matrices provided by the user, which are taken as inputs to define the objective function (deviance function). Once the objective function has been defined, the optimization module is used to optimize the objective function and provide REML estimates of the parameters of interest. Finally, the output module is used to provide an output that can be interpreted by the end user. We developed three main R functions:

• **lmerUvcov:** Fits a linear mixed model with a variance–covariance matrix provided by the user. This function takes as input a formula to specify the response **y**, the fixed effects (fixed) and the random effects (random), a data.frame, and a list (Uvcov) to specify the variance–covariance matrix for random effects. Once the model is fitted, the routine returns an object of class merMod for which many methods are available in R for further processing (e.g., summary, print, predict, and VarCorr).
• **ranefUvcov:** Extracts the conditional means of random effects. This function takes as input an object returned by the lmerUvcov function. If the ranef function in the lme4 package is used taking as input the object provided by the lmerUvcov function, it will extract the conditional means for the random effects in model (6); the conditional means for random effects in model (5) are obtained as explained in the BLUPs section. The ranef function in lme4 is overwritten with ranefUvcov, so the user can call either of these two routines and obtain the same results.
• **ranefUvcovNew:** Obtains BLUPs for new levels of random effects with user-specified variance–covariance matrices. The function takes as input an object provided by the lmerUvcov function and a two-level list with variance–covariance matrices that contains information of the genotype identifiers (GIDs) to be predicted and those that were included when fitting the model. The BLUPs are obtained using partitions similar to those used to derive equation (4).

The software is available in the github repository^1^.

## Examples

In this section, we illustrate the use of the R-package lme4GS with several examples using sample data included in the package. In our examples, we consider only the prediction of random effects and the estimation of variance parameters, although the package is also able to estimate fixed effects.

### Example 1: Genome-Wide Prediction Using Markers and Pedigree

In this example, we analyze a set of 599 wheat lines developed by the CIMMYT Global Wheat Breeding Program. The dataset has been analyzed several times in the literature (e.g., de los Campos et al., 2009; Crossa et al., 2010; Pérez et al., 2010). The dataset includes grain yield information, a pedigree, and 1,477 markers generated by Triticarte Pty., Ltd. (Canberra, Australia^2^). Here, we present the raw phenotypic data, including the replicates in each environment and the pedigree information, in order to show how to use R tools to obtain the additive relationship matrix that is later used as input for fitting the models. The dataset is loaded into the R environment with the commands shown in Box 1.

**Box 1. Loading wheat data.**

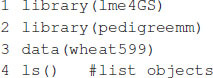

Once the commands are executed, the following objects are available:

• wheat.Pheno: A data.frame with four columns: Env for environments, Rep for replicates, GID for genotype identifiers, and Yield for grain yield.
• wheat.Pedigree: A data.frame with three columns: gpid1 and gpid2, which correspond to the GID of parents 1 and 2, respectively, and progeny, which correspond to the GIDs of progeny.
• wheat.X: A matrix of dimensions 599 × 1,279, which corresponds to Diversity Array Technology (DArT) markers coded as 0 and 1.

A linear model to predict grain yield in one of the environments using markers and pedigree is given by:

(6) $$\text{y}=1\mu +{\text{Z}}_{1}{\text{u}}_{1}+{\text{Z}}_{2}{\text{u}}_{2}+\text{e},$$

where, **y** is the response vector in one environment, **1** is a vector of ones, μ is an intercept, ${\text{u}}_{1}∼MN\left(0,\sigma_{m}^{2}\text{G}\right)$, **G** = **WW**′/*p* (see Lopez-Cruz et al., 2015) is a genomic relationship matrix, **W** is the matrix of markers centered and standardized, *p* is the number of markers, $\sigma_{m}^{2}$ is a variance parameter associated with markers, ${\text{u}}_{2}∼MN\left(0,\sigma_{a}^{2}\text{A}\right)$, **A** is an additive relationship matrix derived from pedigree, $\sigma_{a}^{2}$ is its associated variance parameter, **Z**_1_, **Z**_2_ are matrices that connect phenotypes with genotypes, and **e** is a random term distributed as in model (1). The additive relationship matrix **A** can be easily computed in R using the pedigreemm package (Vazquez et al., 2010); the corresponding Cholesky decomposition can be computed very efficiently, and the package is able to store the result as a sparse matrix. The code in Box 2 computes the **A** and **G** matrices and then fits the mixed model using the lmerUvcov function. After that, it extracts the BLUPs using the ranefUvcov function.

The model fitting time is about 81 s on a computer with a 2.8-GHz Intel Core i7 processor. After the model is fitted, the summary function can be used to show some of the results. The estimates of variance parameters are ${\hat{\sigma }}_{m}^{2}=0.2218$, ${\hat{\sigma }}_{a}^{2}=0.2113,$ and ${\hat{\sigma }}_{e}^{2}=0.0349$ (see Box 3). The functions predict, residuals, etc., that are routinely used after fitting the model with the lmer function; they can also be used with the resulting object.

**Box 2. Computing A and G matrices.**

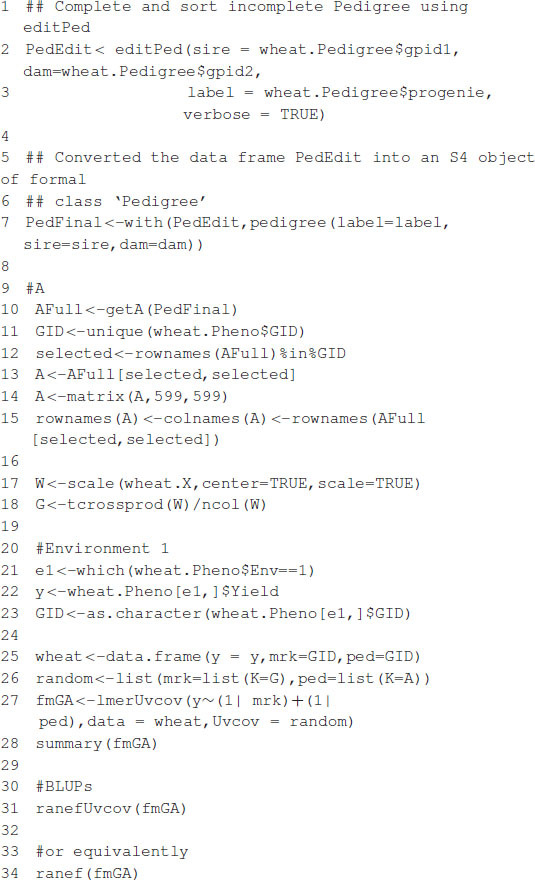

**Box 3. Partial output from Box 2.**

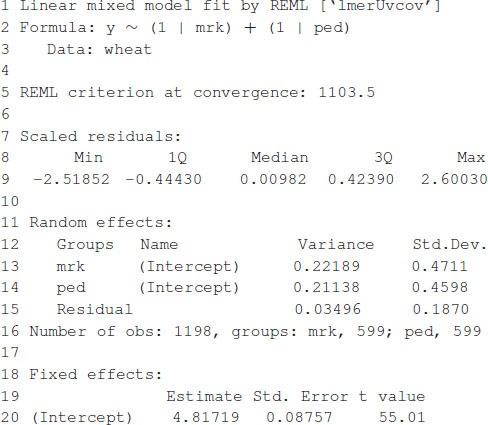

### Example 2: Training and Testing Sets

**FIGURE 1:**
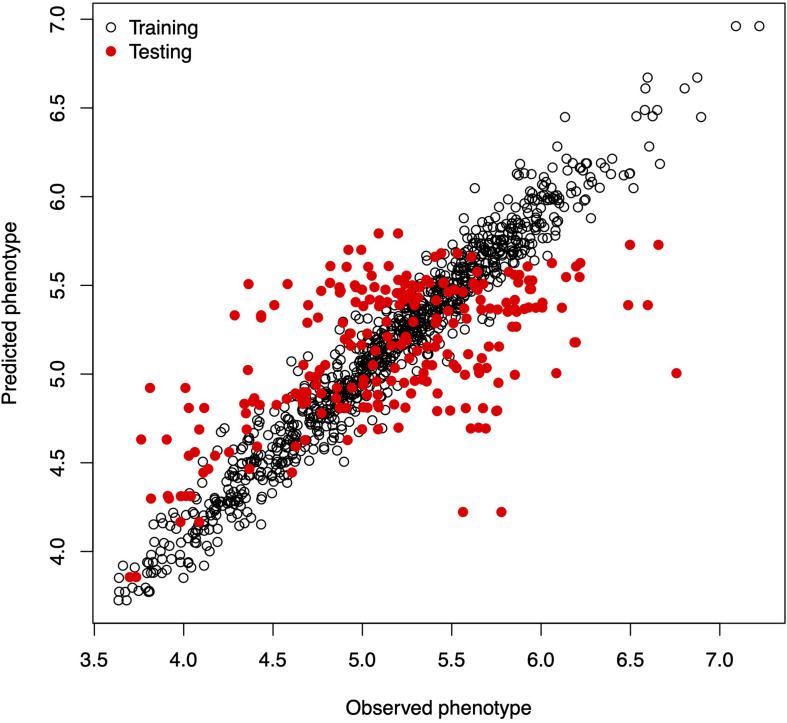
Observed vs. predicted phenotypic values in the training and testing sets.

In this example, we mimic the GS problem faced by breeders; we evaluate the predictive ability of model (6) by CV, which requires randomly partitioning the data into two disjoint sets, assigning 80% of the lines to the training set and the remaining 20% to the testing set. The code in Box 4A partitions the data into the training and testing sets and defines two vectors, y_trn and y_tst, with the phenotypic values of both sets. Next, it creates a list object with the random effects for the linear mixed model. The linear mixed model is fitted using the training set of the data, with the lmerUvcov function. In the next step, we define a list of random effects including the variance–covariance matrices **G** and **A** and the GIDs of the lines to be predicted; the row and column names of the covariance matrices correspond to the GIDs. The ranefUvcovNew function is used for prediction and provides a list of BLUPs for each of the random terms as a result. Finally, the predictions for individuals in the testing set are obtained by simply adding up the intercepts to the BLUPs. Observed and predicted values are stored in a data.frame with three columns: GID, y (observed phenotypic values), and yHat (predicted phenotypic values) used for graphical displays. Figure 1 shows a scatter plot with observed and predicted phenotypic values in both the training and testing sets. Pearson’s correlation coefficient between the observed and predicted values is 0.5638, and the mean squared error (MSE) is 0.2581.

**Box 4A. Single training and testing partition.**

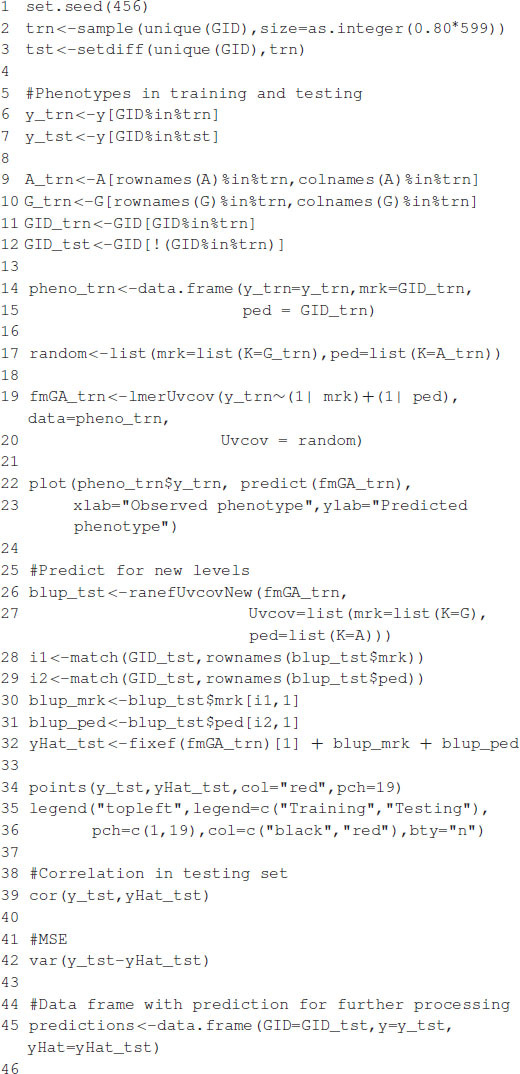

Box 4B shows the R code to perform a five-fold CV that is widely used to study prediction accuracy (e.g., Crossa et al., 2010). We randomly divided the data into five disjoint sets based on the GID, {*S*_1_, …, *S*_5_}. Each set is used to measure prediction accuracy. With the use of these sets, the data are divided into the training and testing populations; for example, the data in {*S*_2_, …, *S*_5_} are the training data, and *S*_1_ are the testing data. The model is fitted using the training data, then phenotypes for *S*_1_ are predicted, and prediction accuracy is measured. The same exercise can be carried out taking *S_f_* as the testing data, *f* = 2, …, 5. Table 1 shows the results of CV, column 1 corresponds to fold, column 2 shows Pearson’s correlation coefficient between observed and predicted values for individuals in the training set, column 3 corresponds to the MSE in the training set, and columns 4 and 5 show the correlations and MSE for individuals in the testing set. The average correlation in the training set is 0.9768, whereas, the correlation in the testing set is 0.5192. The average MSE in the training set is 0.0187, and that in the testing set is 0.2897. The results are as expected: the correlation in the training set is higher than in the testing set, and the MSE is higher in the testing set than in the training set.

**Box 4B. Cross-validation.**

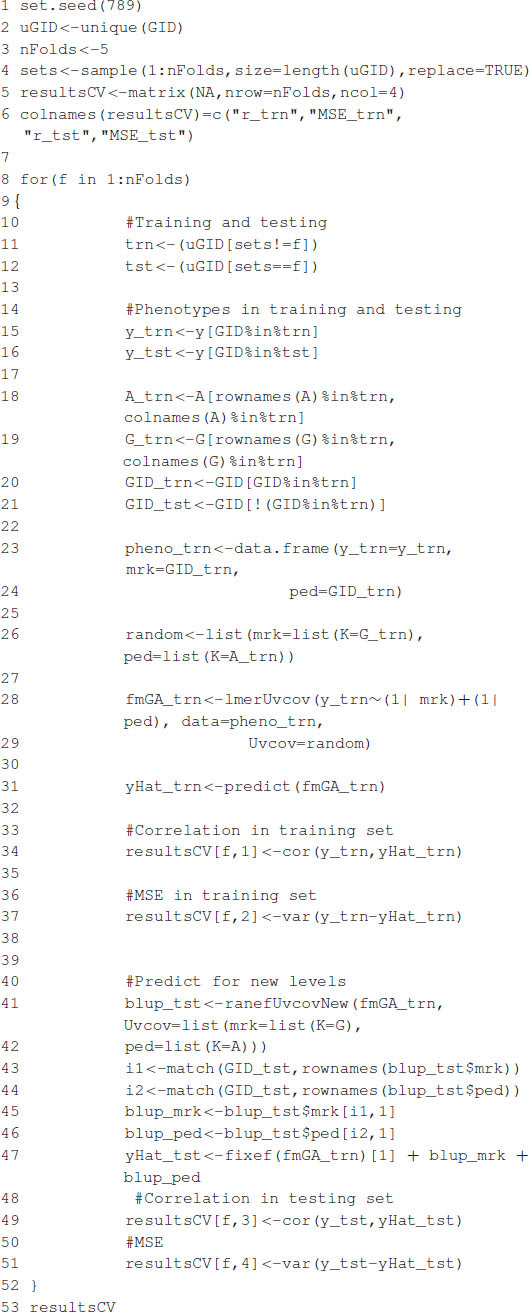

**TABLE 1:** Results from five-fold cross-validation.

Fold	Training		Testing	
	*r*	MSE	*r*	MSE
1	0.9752	0.0201	0.5290	0.2778
2	0.9775	0.0181	0.5680	0.2729
3	0.9755	0.0197	0.5096	0.3035
4	0.9786	0.0173	0.4179	0.3280
5	0.9775	0.0182	0.5714	0.2663
avg	0.9769	0.0187	0.5192	0.2897
sd	0.0015	0.0012	0.0624	0.0256

*MSE, mean squared error.*

### Example 3: Hybrid Prediction

The prediction of hybrid performance is very important in agricultural breeding programs. Technow et al. (2014) and Acosta-Pech et al. (2017) employed G-BLUP type models to predict the performance of maize hybrids. The linear model used to that end is given by:

(7) $$\text{y}=1\mu +\text{W}\theta +{\text{Z}}_{1}{\text{u}}_{1}+{\text{Z}}_{2}{\text{u}}_{2}+{\text{Z}}_{3}{\text{u}}_{3}+\text{e},$$

where, **y** is the response vector; **1** is a vector of ones; μ is an intercept; **W** is the design matrix for environments; θ is the vector of environmental effects (fixed); **Z**_1_, **Z**_2_, and **Z**_3_ are incidence matrices for paternal, maternal, and hybrids, respectively; **u**_1_ and **u**_2_ are vectors of general combining abilities for parental and maternal lines, respectively; ${\text{u}}_{1}∼MN\left(0,\sigma_{1}^{2}{\text{K}}_{1}\right)$, ${\text{u}}_{2}∼MN\left(0,\sigma_{2}^{2}{\text{K}}_{2}\right)$ with **K**_1_ and **K**_2_ relationship matrices for paternal and maternal lines $\sigma_{1}^{2}$, $\sigma_{2}^{2}$ associated variance parameters, ${\text{u}}_{3}∼MN\left(0,\sigma_{3}^{2}{\text{K}}_{3}\right)$, with **K**_3_ = **K**_1_⊗**K**_2_, $\sigma_{3}^{2}$ variance parameter associated with hybrids, and $\text{e}∼MN\left(0,\sigma_{e}^{2}\text{I}\right)$. Note that model (7) can be rewritten as **y** = **X**β + **Z**_1_**u**_1_ + **Z**_2_**u**_2_ + **Z**_3_**u**_3_ + **e**, where, **X** = [**1W**] and β = (μ,θ′)′, which corresponds to model (4) discussed before. To exemplify how to fit this model in the lme4GS package, we used the DT_cornHybrids dataset included in the R-package sommer (Covarrubias-Pazaran, 2016), and we included a copy of the original data in the package (cornHybrids). The dataset contains phenotypic data for grain yield and plant height for 100 out of 400 possible crosses that originated from 40 inbred lines belonging to two heterotic groups, with 20 lines in each. Only 100 hybrids were evaluated in four locations, and then the problem was to estimate their general combining abilities and specific combining abilities and to predict the performance of untested hybrids at each location. The dataset can be loaded in R using the commands shown in Box 5:

**Box 5. Loading maize data.**

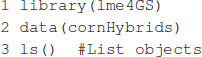

The dataset contains the following R objects:

• maize.Pheno: A data.frame with six columns: Location, GCA1 (Parent 1), GCA2 (Parent 2), SCA (hybrid), Yield, and PlantHeight. Records with missing values in the last two columns correspond to hybrids (identified with the Parent 1:Parent 2 label) that were not evaluated in the field and that we need to predict.
• maize.G: A matrix with relationships between individuals for parents of both heterotic groups (**K**_1_ and **K**_2_). The matrix was computed using 511 single-nucleotide polymorphisms (SNPs) using the A.mat function included in the rrBLUP package (Endelman, 2011). The row names and column names of this matrix correspond to the GIDs for Parent 1 and Parent 2.

The code in Box 6 computes matrices **K**_1_, **K**_2_, and **K**_3_ in model (7) and fits the model using the lmerUvcov function using only observed phenotypic values for plant height.

**Box 6. Fitting model for hybrid prediction.**

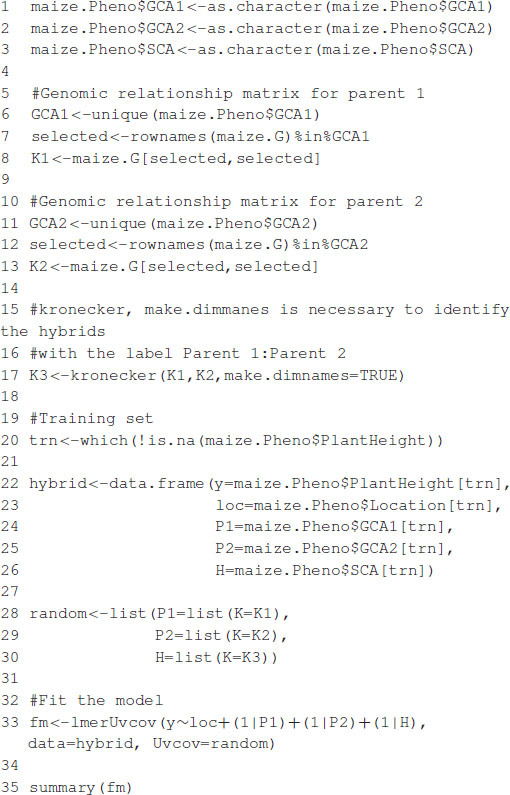

The model fitting takes about 1 s to complete on a computer with a 2.8-GHz Intel Core i7 processor. Once the model is fitted, the summary function can be used to display some relevant information. The summary output is displayed in Box 7, which shows estimates for general combining ability, and specific combining ability and the variance parameter associated with residuals, ${\hat{\sigma }}_{1}^{2}=0.016385$, ${\hat{\sigma }}_{2}^{2}=0.000841$, ${\hat{\sigma }}_{3}^{2}=0.002047,$ and ${\hat{\sigma }}_{e}^{2}=0.001182$.

**Box 7. Output from Box 6.**

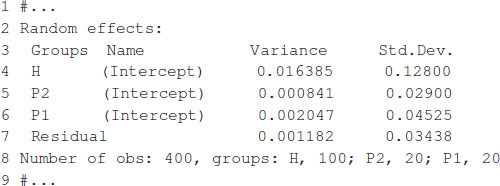

The expected hybrid performance of individuals not evaluated in field can be obtained by combining the outputs from the ranefUvcov and ranefUvcovNew functions. Box 8 shows the instructions to compute the BLUPs for the specific combining ability of hybrids. The ranefUVcov function is called internally in ranefUvcovNew. Box 8 also shows how to extract variance parameters using the VarCorr function and then compute heritability using the results. Following Covarrubias-Pazaran (2016), $h^{2}=\left(\sigma_{1}^{2}+\sigma_{2}^{2}\right)/\left(\sigma_{1}^{2}+\sigma_{2}^{2}+\sigma_{e}^{2}\right)$, which leads to an estimated heritability of 0.70.

**Box 8. Predicting hybrid’s performance.**

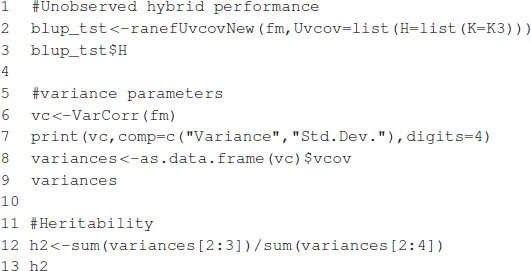

### Example 4: Selection of the Bandwidth Parameter With a Gaussian Kernel

Gianola et al. (2006) introduced the Gaussian kernel into quantitative genetics with the idea of capturing the total genetic effects in the problem of genomic prediction. The Gaussian kernel is defined as (e.g., Morota and Gianola, 2014; Pérez and de los Campos, 2014)

(8) $$\text{K}\left({\text{x}}_{i},{\text{x}}_{j}\right)=\exp\left\{-\theta \frac{d_{ij}^{2}}{m}\right\}=\text{exp}\left\{-\theta \frac{\sum_{k=1}^{m}{\left(x_{ik}-x_{jk}\right)}^{2}}{m}\right\},$$

where, θ is a positive bandwidth parameter; *d*_*ij*_ is the Euclidean distance; and **x**_*ik*_ (*i*, *j* = 1, …, *n*, *k* = 1, …, *m*) is the marker genotype code for individual *i* at marker *k*, and *m* is the number of markers. The bandwidth parameter may be chosen by CV, REML, or maximum likelihood or with Bayesian methods. The Gaussian kernel has been used by many authors for genomic prediction (e.g., de los Campos et al., 2010; Endelman, 2011; Pérez-Elizalde et al., 2015). The selection of the bandwidth is not an easy problem due to high computational cost; de los Campos et al. (2010) and Endelman (2011) proposed evaluating the performance of the model, which includes the Gaussian kernel over a grid of values of θ. Given that θ > 0, if we set ρ = exp(−θ), then ρ ∈ (0,1), so we can define a grid of values for ρ and then, using these values, set the values for θ, that is, θ = −log ρ, so that equation (8) can be rewritten as $\text{K}\left({\text{x}}_{i},{\text{x}}_{j}\right)=\exp\left\{\log\rho d_{ij}^{2}/m\right\}$. Another kernel that is also used in genomic prediction is the exponential kernel (e.g., Piepho, 2009; Endelman, 2011):

(9) $$\text{K}\left({\text{x}}_{i},{\text{x}}_{j}\right)=\exp\left\{-\theta d_{ij}/\sqrt{m}\right\},$$

where, all the terms have been described previously. Similar to the case of the Gaussian kernel, the model can be reparametrized in terms of parameter ρ ∈ (0, 1).

We developed the function theta_optim that fits model (5) when one of the random terms (**u**_*j*_, *j* = 1, …, *q*) includes as the variance–covariance matrix a Gaussian or exponential kernel. This function takes as input the same objects as the lmerUvcov function and a list (kernel) containing (i) a matrix with distances $\left(\left\{d_{ij}/\sqrt{m}\right\},i,j=1,\ldots ,n\right)$ or the marker matrix ({*x*_*ij*_}, *i* = 1, …, *n*, *j* = 1, …, *m*), (ii) the kernel type (either “gaussian” or “exponential”), and (iii) a sequence of values for θ; the IDs for the individuals are taken directly from the row names of matrices that provide the distances or the markers. If the sequence of values for θ is not provided, then it is generated automatically. The software then fits the mixed model in (5) using the lmerUvcov function for each of the distinct values of θ. The value of θ that maximizes the log-likelihood is chosen as the optimum. The function returns a list with the following elements: a vector of values of the log-likelihood, the maximum value of the log-likelihood, the values of θ used for fitting the model, the optimum value of θ, the fitted model, and the kernel computed with the optimum value of θ.

In the following example, we show how to predict grain yield using a relationship matrix derived from a Gaussian or exponential kernel and a relationship matrix derived from a pedigree. A linear model to predict grain yield for environment one is analogous to model (1):

(10) $$\text{y}=1\mu +{\text{Z}}_{1}{\text{u}}_{1}+{\text{Z}}_{2}{\text{u}}_{2}+\text{e},$$

where, **y** is the grain yield; **1** is a vector of ones; μ is an intercept, ${\text{u}}_{1}∼MN\left(0,\sigma_{m}^{2}\text{K}\right)$, with **K** a kernel, which can be either Gaussian or exponential, and $\sigma_{m}^{2}$ is a variance parameter associated with markers; ${\text{u}}_{2}∼MN\left(0,\sigma_{a}^{2}\text{A}\right)$, where, **A** is an additive relationship matrix derived from pedigree, and $\sigma_{a}^{2}$ its associated variance parameter; **Z**_1_, **Z**_2_ are matrices that connect phenotypes with genotypes; and **e** is a random term distributed as in model (1).

**FIGURE 2:**
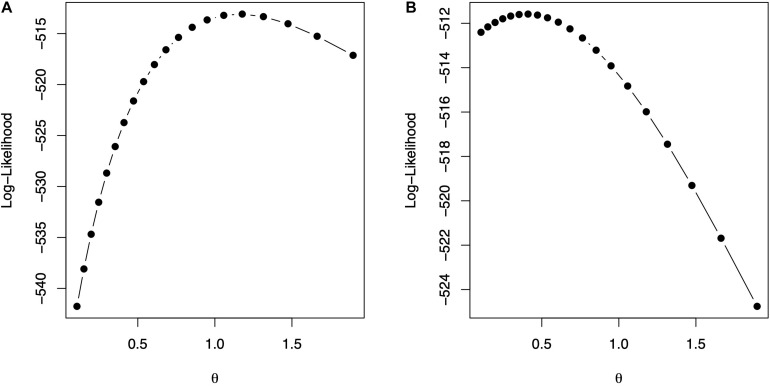
The values of the bandwidth parameter vs. the log-likelihood. **(A)** Gaussian kernel. **(B)** Exponential kernel.

The code in Box 9 is used to fit model (10) for Gaussian and exponential kernels. Figure 2 shows the profile of the log-likelihood for different θ values. For the Gaussian kernel, the maximum of the log-likelihood is equal to -513.0865, attained at $\hat{\theta }=1.1779$, whereas, for the exponential kernel, the maximum of the log-likelihood is equal to -511.585, attained at $\hat{\theta }=0.4107$. The code in Box 10 shows how to summarize parameter estimates for the fitted model with the optimum value of the bandwidths from, where, estimates of the variance parameters can be obtained. The model fitting time is about 1,608 s for the model with Gaussian kernel and 1,701 s for the model with exponential kernel using the same processor described before. Note that the selection of bandwidth parameter is a very computer intensive task, but several authors (e.g., Endelman, 2011; Pérez-Elizalde et al., 2015) have reported that the prediction accuracy with nonadditive kernels is higher than the prediction accuracy of ridge regression (or equivalently GBLUP).

**Box 9. Gaussian and exponential kernel.**

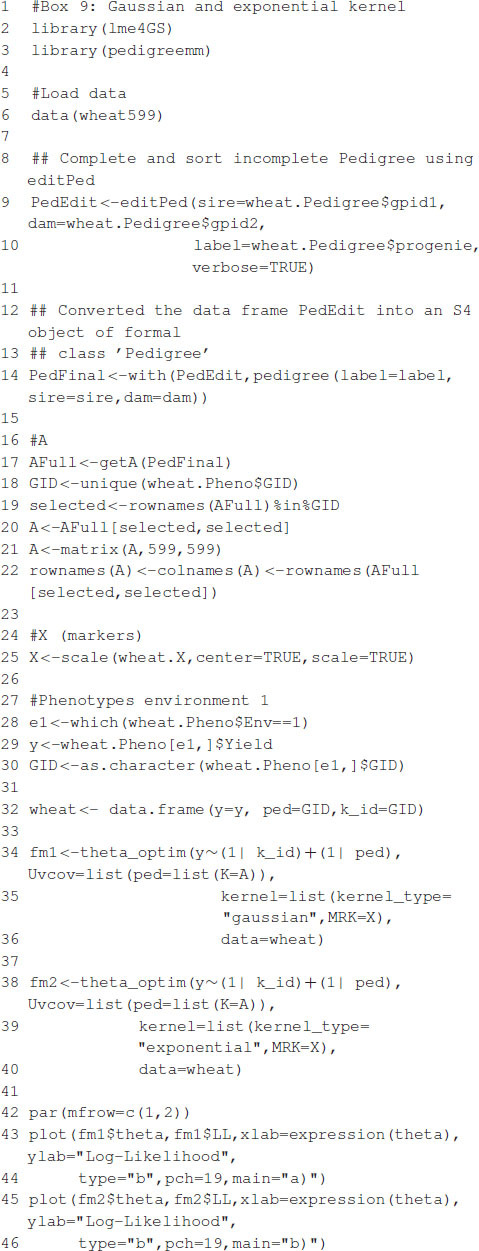

**Box 10. Summary of fitted models.**

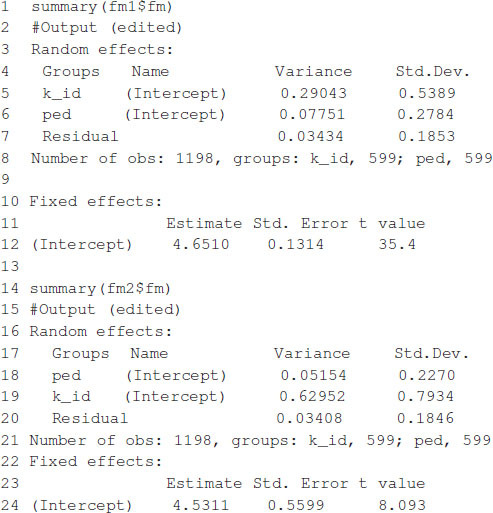

### Computational Times and Comparison With Other Software

We fitted models (6) and (7) in sommer (Covarrubias-Pazaran, 2016) and BGLR (Pérez and de los Campos, 2014). In the case of BGLR, the number of iterations for the Gibbs sampler was set to 30,000. We were unable to fit the models in rrBLUP (Endelman, 2011) because it is not possible to include more than one covariance matrix in the software; that is also the reason that we were unable to fit model (10) with this software. The predictions from the different software programs were about the same. Here, we present a small comparison of running times for model (6) fitted in Box 2, model (7) fitted in Box 6, and model (10) fitted in Box 9 with Gaussian and exponential kernels. Covarrubias-Pazaran (2016) also included a benchmark of sommer against other packages. Models were fitted using a 2.8-GHz Intel Core i7 processor in R-4.0.5 (R Core Team, 2021). Table 2 presents the resulting time (in seconds) it takes to fit the different models. Some entries in the Table 2 are empty because the corresponding models cannot be fitted in the corresponding software package. From this Table 2, we conclude that sommer is the fastest software, followed by lme4GS and BGLR.

**TABLE 2:** Time comparison (seconds) among different software for models fitted in the work.

Software	Version	Examples			
		Model (6)	Model (7)	Model (10) Gaussian	Model (10) exponential
lme4GS	0.1	81.5	1.3	1,608.8	1,701.1
BGLR 0.8	0.8	143.0	20.2	–	–
sommer 4.1.3	4.1.3	46.0	2.7	–	–

## Conclusion

We developed an R software package that can be used to fit mixed models with user-defined covariance structures for random effects. The software was developed with applications of GS in mind, mainly for applications in plant breeding with small to moderately sized datasets. However, given the omnipresence of mixed models, the package can be used in other research areas. The software fits the model using well-known and widely tested computational routines available in the lme4 package. The software provides a user-friendly and intuitive interface that allows users to fit a wide variety of classic linear mixed models.

## Data Availability Statement

Publicly available datasets were analyzed in this study. This data can be found here: https://github.com/perpdgo/lme4GS.

## Author Contributions

DC-P, PP-R, and JC conceived the work and wrote the manuscript. DC-P and PP-R wrote the software. CV-C, SP-E, and MV-P assisted in drafting the manuscript, discussed the analysis, and provided useful comments. All authors contributed to the article and approved the submitted version.

## Conflict of Interest

The authors declare that the research was conducted in the absence of any commercial or financial relationships that could be construed as a potential conflict of interest.

## Footnotes

1 https://github.com/perpdgo/lme4GS

2 https://www.diversityarrays.com

## Abbreviations

BGLR: Bayesian generalized linear regression
BLR: Bayesian linear regression
BLUP: best linear unbiased prediction
GEBV: genomic estimated breeding value
GLMM: generalized linear mixed model
GS: genomic selection
LMM: linear mixed model
REML: restricted maximum likelihood.
